# A Review of Electro Conductive Textiles Utilizing the Dip-Coating Technique: Their Functionality, Durability and Sustainability

**DOI:** 10.3390/polym14214713

**Published:** 2022-11-03

**Authors:** Alenka Ojstršek, Laura Jug, Olivija Plohl

**Affiliations:** Faculty of Mechanical Engineering, University of Maribor, Smetanova 17, 2000 Maribor, Slovenia

**Keywords:** electro conductive textiles, dip-coating, characterization, functional features, durability, sustainability, recyclability

## Abstract

The presented review summarizes recent studies in the field of electro conductive textiles as an essential part of lightweight and flexible textile-based electronics (so called e-textiles), with the main focus on a relatively simple and low-cost dip-coating technique that can easily be integrated into an existing textile finishing plant. Herein, numerous electro conductive compounds are discussed, including intrinsically conductive polymers, carbon-based materials, metal, and metal-based nanomaterials, as well as their combinations, with their advantages and drawbacks in contributing to the sectors of healthcare, military, security, fitness, entertainment, environmental, and fashion, for applications such as energy harvesting, energy storage, real-time health and human motion monitoring, personal thermal management, Electromagnetic Interference (EMI) shielding, wireless communication, light emitting, tracking, etc. The greatest challenge is related to the wash and wear durability of the conductive compounds and their unreduced performance during the textiles’ lifetimes, which includes the action of water, high temperature, detergents, mechanical forces, repeated bending, rubbing, sweat, etc. Besides electrical conductivity, the applied compounds also influence the physical-mechanical, optical, morphological, and comfort properties of textiles, depending on the type and concentration of the compound, the number of applied layers, the process parameters, as well as additional protective coatings. Finally, the sustainability and end-of-life of e-textiles are critically discussed in terms of the circular economy and eco-design, since these aspects are mainly neglected, although e-textile’ waste could become a huge problem in the future when their mass production starts.

## 1. Introduction

As technology becomes increasingly mobile, the next step is to integrate devices and advanced functionalities into flexible textile substrates, which, when combined with different solutions, e.g., the Internet of Things (IoT), data analysis (big data) and Artificial Intelligence (AI), provide a wide range of actions, such as virtual reality, device-to-device communications, and cyber-physical systems [1] with a strong impact on the 4th industrial revolution [2]. Therefore, textiles that were traditionally considered as a protective material against environmental impacts now play a crucial role in wearable electronics (smart, textile-based devices/electronic textiles), contributing to the areas of energy harvesting, energy storage, sensors, real-time monitoring of healthcare, personal thermal management, and even aerospace-grade smart textiles [3,4]. Electro conductive textile (fibers, yarn, or fabric) with its mechanical flexibility, long-term durability, and stability under harsh conditions, is an essential part of flexible and stretchable textile-based electronics, and, herein, the integration/application technique of conductive compounds influences textiles’ electrical properties significantly, as well as the wash/wear durability of the final product [2,5,6].

The overall conductive textiles market is projected to register over 16.3% CAGR, to reach around USD 5.4 billion during the forecast years (2020–2027), highlighting the importance of studies on conductive textiles. The key factors driving market growth are the military and defense industries, the increasing adoption of smart fabrics in numerous applications, and high demand for health-monitoring wearables. Some other applications include conductive textiles for astronauts, flexible energy storage, biological chemistry, and protection against weathering [7]. Additionally, the easy recycling of these textiles is expected to influence the ’industry’s growth positively. 

**Figure 1:**
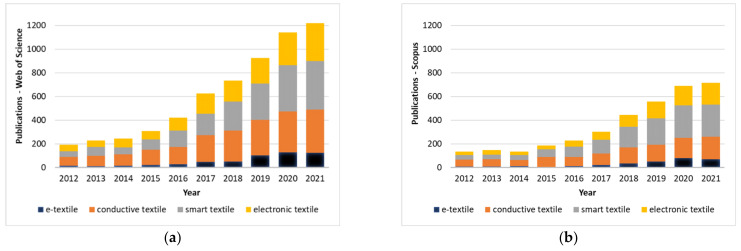
Published scientific articles from 2012 up to 2021 using four keywords: “e-textile”, “conductive textile”, “smart textile” and “electronic textile”: (**a**) Web of Science; and (**b**) Scopus.

Simultaneously with the enormous growth of the conductive textiles’ market, the number of scientific papers published in the last 10 years in this field has increased, as can be seen in Figure 1. The queries in Web of Science (Figure 1a) and Scopus (Figure 1b) show the biggest jump in the number of research papers for the last 4 years. Although there is a slight decline observed for the year 2021, the average growth trend indicates the increased relevance of the presented research topic. From four selected keywords (“e-textile”, “conductive textile”, “smart textile” and “electronic textile”), the keyword “smart textile” is the most relevant term, as expected, due to the growing popularity of the so-called Internet of Things (IoT) and Internet of Bodies (IoB).

Table 1 summarizes the number of European projects related to the article topic under the sixth and seventh framework program for research and technological development (FP6 and FP7), and Horizon 2020 EU’s research and innovation funding program 2014–2020 (H2020) from the Community Research and Development International Service (CORDIS). Different key words and their combinations were employed therein. The results show a rising number of completed research projects, which indicates an increased interest to invest in the developing sector of so-called wearable electronics. The key word “e-textile”, which is the short name for an electronic textile system (based on conductive textiles), seems to be the most relevant term, since many of the completed projects from the FP6, FP7 and H2020 were found under it.

This article presents an in-depth study of the current state-of-the-art in the field of electro conductive fiber-forming materials utilizing a relatively simple and low-cost dip-coating process, which can easily be up-scaled and integrated into a conventional textile processing plant. The main contribution of this review focuses on the application of different electro conductive compounds, such as electro conductive polymers, carbon-based materials, metal, and metal-based nanomaterials, as well as their combinations, and their advantages and disadvantages. Emphasis is also given to the characterization techniques of coated electro conductive materials, including electrical, physical-mechanical, chemical, morphological, and comfort properties. Since it is of great importance to combine high electrical conductivity of textiles with advanced functional properties, recent studies on multifunctional conductive textiles were discussed. The last two sections are dedicated to two important, but nearly neglected fields with a lack of proper standardized procedures when dealing with conductive textiles; i.e., wash and wear durability, and sustainability/recyclability.

## 2. Dip-Coating to Obtain Electro Conductive Textiles

**Figure 2:**
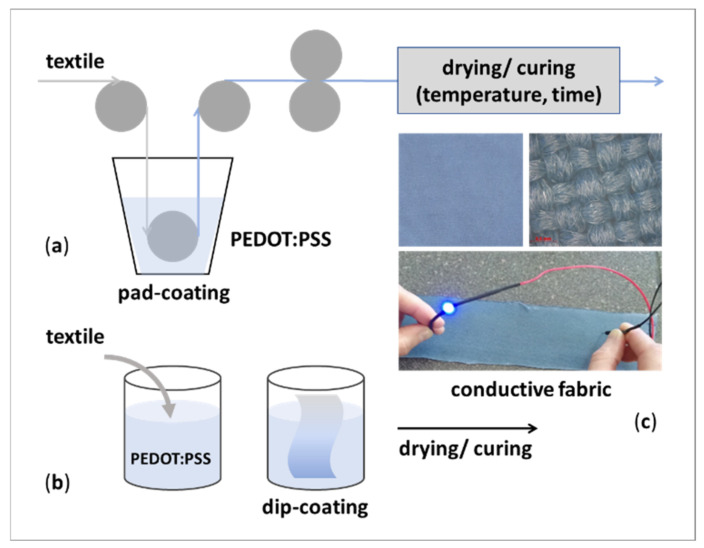
Schematic presentation of: (**a**) A continuous coating process; (**b**) A discontinuous coating process; and (**c**) A photo and optical microscopic image of poly-3,4-ethylenedioxythiphene: polystyrene sulfonate (PEDOD:PSS)-coated cotton fabric, and quick bulb testing of its electrical conductivity.

Dip-coating (or pad-coating) is a the relatively simple, low-cost and scalable, and, thus, widely used fabrication techniques to apply conductive (nano) materials onto fibers, yarns, or fabrics, as it does not require any special or expensive equipment [6,8]. The entire process can be continuous, as presented in Figure 2a, or discontinuous (Figure 2b). It involves the immersion of the fibrous substrate for a short period into the respective aqueous-based coating solution, which, in the case of conductive textiles’’ processing, contains different types of conductive compounds; i.e., electro conductive polymers, carbon-based structures, inorganic metallic (nano)structures, or their combinations [9]. Thereby, the conductive compound is deposited on both sides, over the whole surface [10] as seen in Figure 2c.

After depositing the conductive solution onto the fibrous material, the drying phase is carried out at temperatures of 110 °C up to 180 °C for a short time period (3 to 15 min), using hot air or infra-red (IR) heating. The process is also known as a curing, during which the excess solvent evaporates and the conductive (nano)compounds fix/crosslink permanently to the ’textile’s surface, ensuring a stable coating. In fact, the dip/dry cycles can be repeated several times, with the aim to increase the amount of deposited conductive material (the so-called multi-layered dip-coating method). Moreover, the drying temperature versus time should be considered, to prevent damage of the basic textile material [11,12]. 

The main disadvantage of the dip-coating procedure is the variance in the smoothness/roughness of the ’textile’s surface and uniformity of the applied conductive layer(s), affecting the variation in electrical resistance [13]. The thickness of the layers cannot be fully controlled, since it depends on the surface morphology and the tension of the textile substrate [14], as well as on processing parameters such as time, temperature, withdrawal speed, compound concentration, composition of the coating bath, etc. [8].

As mentioned above, numerous electro conductive compounds can be applied to fibers/yarns/fabrics by employing the dip-coating technique. In the following sub-sections, the most commonly used materials are presented, as well as different applications.

### 2.1. Electro Conductive Polymers

Electro conductive polymers are defined as organic materials that show either conductive or semi-conductive properties [15,16]. Polymers themselves are generally nonconductive, however, certain polymers could be modified to become intrinsically or extrinsically conductive [17]. Intrinsic conductance is accomplished by the formation of conjugated double bonds, which are usually combined with an additional doping of a counter ion into the polymer. Electrical conductivity can be enhanced by the addition of a polar organic solvent as the second dopant [18]. An example of such a polymer is poly-3,4-ethylenedioxythiphene (PEDOT), which should be doped with, e.g., polystyrene sulfonate (PSS), creating compounds with free electrons in the conduction band, then allowing the transfer of charges [16]. In contrast to intrinsically conductive polymers, extrinsically conductive polymers are formed by the blending of the insulation polymer matrix with conductive fillers. 

Among the numerous intrinsically conductive polymers, polypyrrole (PPy), polyaniline (PANI) and polythiophenes (PTh) have recently attracted the greatest attention for the fabrication of conductive textiles for diverse applications due to their unique combination of properties, such as electrical conductivity, electromagnetic shielding ability, low weight, bendability, high elasticity, biocompatibility, high transparency, low cost, excellent environmental durability, solution processability, good adhesion to diverse substrates, and ease of preparation and application [18,19,20]. Moreover, conductive polymers do not cause skin irritation or long-term toxicity [21], although they have a limited life cycle due to degradation over time, and a significantly lower conductivity and permittivity compared with metal-based compounds, which results in a relatively long path of electromagnetic waves in the material, as reported by [22,23].

The mechanical properties of conductive polymers depend on their chemical composition and can be changed by the addition of specific compounds, e.g., plasticizers (increased flexibility and resiliency after deformation), or by preparation of polymeric blends [10]. They are especially convenient for roll-to-roll processing, and, thus, compatible with textile finishing technologies such as dyeing and printing [19], with the potential to be used as sensors (amperometric and photometric), antennas, wearable electronics, electromagnetic shielding, displays, radio frequency identification tags, organic light-emitting diodes, interconnections, energy harvesting, storage devices, electrodes for capacitors and batteries, etc. [10,19,21].

One of the most studied and successfully employed conductive polymers for dip-coating of different types and forms of textiles is PEDOT:PSS (Figure 2), as can also be seen in Table 2. Tadesse et al. [24] reported a dip-coating approach more effective for this type of polymer as compared to the screen-printing technique, enabling greater electrical conductivity, resistance to stretching, and cyclic stretching characteristics. These fabrics had a more uniform and smoother surface look, whereas screen-printed fabrics exhibited uneven aggregation. PEDOT:PSS is a polyion complex with excellent thermal and chemical stability, good film-forming properties, a highly reproducible procedure, and can be readily distributed in water as colloidal gel particles with sizes of several tens of nanometers [25]. The characteristic of this complex can be modified by the addition of organic co-solvents (e.g., glycerol, dimethyl sulfoxide (DMSO) sorbitol), surfactants, and/or wetting agents into the aqueous dip-solution mixture, with the aim to increase the polymer’ conductivity and coatability on hydrophobic surfaces, as well as to improve the physical-mechanical features [16]. A disadvantage of PEDOS:PSS is a bluish hue, as can also be seen in Figure 2c, which depends on its concentration in the solution, influencing the coating transparency, and, consecutively, interfering with the textiles’ coloration. A detailed description of its properties and applications can be found in Tseghai et al. [19].

Some of the research is focused on other conductive polymers. Indarit et al. [28] coated commercial yarns from cotton, rayon and polyester with PANI in order to detect the concentration of ammonia gas in the environment or the working area (Table 2). Kim et al. determined electrical conductivity and, further, the radar absorbing ability of a PANI:CSA dip-coated aramid fabric. They found significantly lower conductivity of PANI:CSA-functionalized samples in comparison with PEDOT:PSS-treated, due to the lower coating amount and aggregated morphology. Lv et al. [30] reported superior electrical conductivity of uniformly PPy-coated textile electrodes using cotton, wool, silk and polyester fabrics, without influencing the textiles’ breathability, flexibility, or comfortability.

### 2.2. Carbon-Based Materials

Carbon-based structures such as graphene (G), graphene oxide (GO), and reduced graphene oxide (rGO) and carbon nanotubes (CNTs), are very promising candidates for textiles’ functionalization, due to their light weight, high electrical conductivity and outstanding mechanical features [32]. Among those listed, the most popular compound for dip-coating of different textiles is GO (or rGO), due to its good dispersibility in aqueous solution and greater chemical interactions between the oxygen-based polar groups of GO (carboxyl, carbonyl, hydroxyl, and epoxide) and diverse functional groups of fabric as compared to the pure G [33,34,35]. In addition, the reduction of GO into rGO using Na_2_S_2_O_4_ and L-ascorbic acid as reducing agents can eliminate oxygen-containing functional groups, which leads to enhanced electrical conductivity [36].

**Figure 3:**
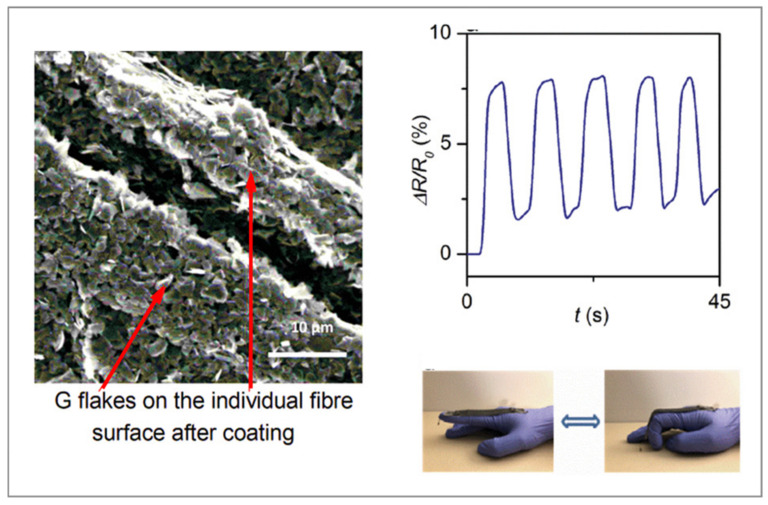
SEM image of G nanosheet coated textiles and corresponding strain sensing measurement (reprinted under the terms of the CC BY Creative Commons Attribution 4.0 International License from Ref. [41] Copyright 2022 John Wiley and Sons).

As can be seen in Table 3, numerous electro conductive textiles were recently fabricated by dip-coating and applying G and its derivates, namely: nylon fabric [37,38,39], Spandex yarn [36,40], cotton fabric/yarn [38,41,42] (Figure 3), wool fabric [43], polyurethane yarn [44], etc. As reported by [40], conductivity increases exponentially with the dipping cycles due to the enlarged thickness of the deposited layers. Moreover, it depends on the solvent used for dispersing G. 

In the study performed by [22], G was used as a filler in waterborne polyurethane (WPU) composites, which was coated on para-aramid knitted fabric with the aim to prepare a textile-based heating element for protective clothing. The G filler formed a percolation threshold in the WPU matrix, inciting the creation of an electrically conductive path that improved the electrical properties. More details about GO-coated commercial textiles for e-textiles’ applications are available in [47].

CNTs are another electro conductive carbon-based material (Table 3), used mainly in the field of wearable energy-related applications [6,42,46], and for strain sensing [6,36]. CNTs are tubular carbon allotropes that resemble graphene sheet rolls. They are classified into two groups based on the number of layers and the manufacturing process, namely, single-walled carbon nanotubes (SWCNTs) and multi-walled carbon nanotubes (MWCNTs). CNTs are known for their outstanding mechanical properties including high elastic modulus and strength, as well as excellent optical, thermal, and electrical properties [48]. In a study performed by Xu et al. [36], two different types of carbon-based materials were combined, i.e., MWCNTs and two rGOs for a functional coating of spandex fabric to develop micro-folded structures between two layers of rGO, which results in good linearity. In addition, the durability of the modified fabric was increased by strengthening the connections between the conductive layer and the fabric surface through covalent immobilization.

Despite significant improvement in the sheet resistance of carbon-based nanomaterials compared with the conductive polymers, the use of CNTs for large scale applications is still complicated due to their controlled structure [13]. It has also been proven that CNTs can cause various adverse health effects, such as pneumonia, and MWCNTs have been identified as potentially carcinogenic [49]. Therefore, some precautions should be considered since conductive textiles coated with these types of compounds can also be in close contact with the skin.

### 2.3. Metals and Metal-Based Materials

Metal and metal-based compounds are the next group of electro conductive materials suitable for dip-coating of textiles, although reports on their deposition on textile fibers/fabrics via low-cost dip coating methods are rather scarce [50,51]. Some other processes are used more frequently for textiles’ metallization, as reviewed by [52]. Dip-coating by metal nanoparticles (NPs) or nanowires (NWs) gives superior electrical conductivity compared with other conductive compounds, although thick metal layer(s) on the fibrous surface could lead to reduced flexibility and bendability of the textiles, and, consequently, make them less comfortable for wearing due to the rigidity of the metals. Thin nanometal films and NWs, which keep the pliability of the fibers while still retaining their good conductivity, could be a solution to these problems [53]. Generally, there are three types of nanoscopic metal materials: zero-, one- and two-dimensional (0D, 1D and 2D). 0D NPs and 1D NWs are the most commonly used compounds to attain the electro conductive functionalization of textiles, reducing the cost, volume, and weight while improving the physical-mechanical and comfort features of the final products [35,54]. 

Among the different metal-based nanomaterials appropriate for preparation of e-textiles, silver (Ag) NPs and Ag NWs are the most studied compounds, due to their high bulk electrical conductivity (6.3 × 10^5^ S/cm), good optical and thermal properties, superior resistance to oxidation, antibacterial property, and simple application from an aqueous solution [50,54]. Zhang et al. [51] reported the application of 7.5 wt% Ag NWs on cotton fabric, which was pre-treated with a 24.2 wt% of polyethylenimine/phytic acid layer, with the aim to intensify the electrostatic interaction and the hydrogen bonding effect. The designed fabric had a high electrical conductivity of 24.16 × 10^2^ S/m and an impressive EMI shielding effectiveness of 32.98 dB, as well as an effective self-extinguishing effect, even after washing. Similar research was provided by Chen et al. [55], who dip-coated pre-modified PEI-cotton fabric with Ag NWs, achieving a low electrical resistance of 2.4 Ω, as well as good durability and flexibility, and, consecutively, they successfully prepared personal heating protective materials. Lian et al. [50] suggested a multifunctional integration strategy employing multilayers of dip-coated Ag NWs fabric, which, simultaneously, acts as a pressure sensor in combination with Joule heating, radiative warming, and PM 2.5 filtration.

Since the use of Ag nanomaterials is limited by their high cost and availability, copper (Cu) nano-compounds have been studied intensively recently as a substitute for Ag, because of their low price, relative ease of synthesis and processing, strong mechanical features, comparable bulk electrical conductivity (5.96 × 10^5^ S/cm), and considerable resistance to oxidation after the application of protective coatings [56]. Ye at al. [57] performed a two-step dip-coating method to coat Cu onto fabric made of 80% polyester and 20% cotton, obtaining a soft 3D Cu-fabric current collector with exceptional long-cycle performance. Mamun et al. [58] described a scalable coating process to develop a durable conductive cotton fabric, where Cu particles are deposited on the fabric surface by continuous dipping and a drying method in metallic salt and a reducing agent (ascorbic acid), reducing possible oxidation, minimizing the costs, and maximizing the conductivity of the coated fabric.

Also, other metal nanomaterials were used as conductive coatings and fillers such as ZnO [59], Au [60], Sn and Al [61]. The instability of metals (risk of oxidation), the variability of costs, poor interfacial adhesion with fabrics, weight, rigidity, low durability during wash and wear, and concerns about their biocompatibility limit their applications [5,52,62]. Moreover, metallic nanostructures could also cause skin irritation and long-term toxicity in contact with the skin, as well as serious health problems, e.g., genotoxicity [21,49].

The interest of researchers in recent years has focused on a new family of 2D transitional metal carbides and nitrides, the so-called MXenes, for the creation of conductive textile-based devices. Due to their unique features, i.e., high electrical conductivity (up to 20,000 S/cm), higher strength and stiffness compared to other solution-processed 2D materials, excellent volumetric capacitance, specific surface area, good hydrophilicity, excellent ion intercalation behavior, scalable solution syntheses (kg batches), sufficient environmental stability, pseudocapacitance and aqueous solution processing, MXenes offer a wide range of possible applications in the field of lightweight and flexible e-textiles, including electrochemical energy storage, catalysis, physiotherapy, Joule heating, strain sensing and EMI shielding [3,23,63,64,65,66].

**Figure 4:**
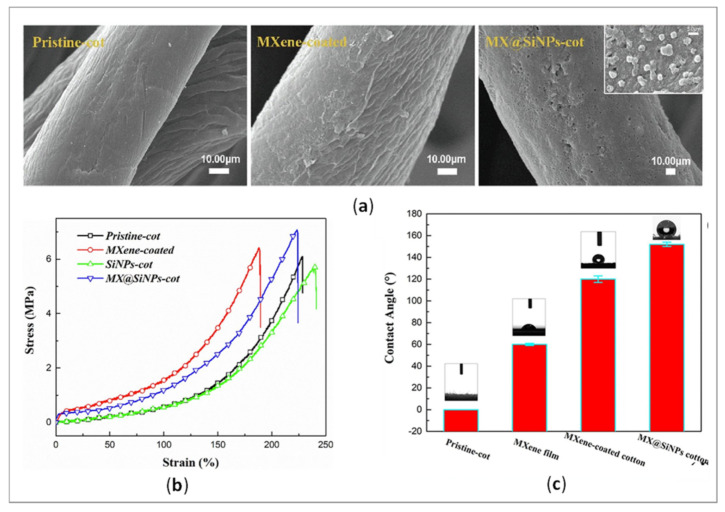
(**a**) SEM images of untreated cotton, MXene-coated and MXene@SiNPs-coated cotton, (**b**) Corresponding stress-strain results, and (**c**) Water Contact Angle (WCA) of samples (reprinted with permission from Ref. [3] Copyright 2022 Elsevier).

Uzun et al. [66] reported a standard dip-coating method for the creation of highly conductive cotton and linen textiles using additive-free 2D Ti_3_C_2_T_x_ flaxes distributed in water for EMI shielding. Wang et al. [3] fabricated a waterproof and wearable multifunctional sensor by integrating 2D MXene nanosheets and Si nanoparticles on a cotton fiber substrate (Figure 4a). In addition to its high performance (strain-stress results; Figure 4b), the sensor retained intrinsic conductivity, even under wet and corrosive conditions, due to its hierarchical structure and the low surface energy of the Si NPs layer with a high contact angle (Figure 4c). In a study performed by Zheng et al. [67], a conductive MXene-functionalized cotton was used for flexible, wearable pressure sensors. The MXene was applied according to a simple dip-coating technique, showing high sensitivity of 5.30 kPa^−1^, a wide sensing range of 0–160 kPa, and a rapid response time of 50 ms/20 ms.

### 2.4. Combinations of Different Types of Conductive Compounds

Diverse combinations of electro conductive compounds and intrinsic conductive polymers were employed with the aim to overcome the major drawback of electro conductive polymers, i.e., low electrical conductivity, as compared to the metal-based compounds, and to enhance the adhesion and comfort properties of metal-coated textiles, as well as to reduce the oxidation of metals. Hwang et al. [68] reported highly conductive silk yarns (up to ~320 S/cm) using a combination of Ag NWs and PEDOT:PSS that remained conductive even after ten cycles of machine washing. Li et al. [25] also used Ag NWs/PEDOT:PSS compounds for multistep dip-coating of aramid non-woven fabric, in order to fabricate high-performance EMI shielding and Joule heating fabric with electrical resistance as low as 0.92 ± 0.06 Ω/sq and good flexibility. The PEDOT:PSS layer, in this case, improved the adhesion between the Ag NWs and the non-woven fabric, as well as enlarged the thermal stability of the fabric. Amirabad et al. [69] prepared thermoelectric polyester/linen fabrics by ultrasonic-assisted dip-coating of polyaniline/graphene nanosheets (PANI/GNS) on a polyester fabric using 0.5, 2.5, 5, and 10 wt% of GNS (the electrical conductivity was enhanced from 0.0188 up to 0.277 S/cm). Alamer [70] applied polyaniline/carbon black (PANI/CB) composite onto cotton fabric, improving the electrical conductivity compared with the pure PANI coating. By enhancing the PANI/CB amount in the sample, the hybrid also moves from the surface of the fabric to the region between the fibers. Therefore, more electrical pathways are created, reducing the sheet resistance. Wu and Hu [71] combined the aqueous PEDOT:PSS solution with MWCNs to improve the conductivity of cotton and polyester yarns, resulting in 13.83 S/m. The authors suggested that as coated yarns can be used as thermoelectric legs in the future design of the fabric TEG. Ahmed et al. [72] applied a combination of rGO and PEDOT:PSS to create a stretchable, thermally stable, and highly conductive cotton textile. Lima et al. [73] fabricated flexible, wearable, multifunctional electronic textiles by interfacial polymerization of PP on CNs/cotton yarn, incorporating antibacterial, as well as good electrochemical and electrical heating features.

Additional combinations are also possible, imparting fabrics’ multi-functionality. Bi et al. [74] fabricated Ni/CNT flexible flax fabric with superior mechanical strength and anti-corrosive properties for multifunctional sensing by the dip-coating of CNT, followed by subsequent electroless Ni deposition, involving PDA surface treatment, Ni activation, and Ni coating. Zheng et al. [67] prepared multifunctional rGO/MXenes decorated cotton fabrics with high electrical conductivity (surface resistance of 13.8 Ω/sq), excellent electrochemical performance, good flexibility, and breathability using facile and scalable dip-coating and spray-coating techniques. Moreover, the rGO/MXene fabrics had good Joule heating and EMI shielding performance, as well as high sensitivity when they are applied as strain sensors to detect human motion.

## 3. Characterization of Conductive Textiles

### 3.1. Electrical Conductivity

Textiles themselves do not conduct electric currents and, thus, they are considered as non-conductive materials [52]. By the application of conductive nanoparticles, polymers and/or compounds on the surface, they become electrically conductive and can be further used for wearable electronic applications. The electrical conductivity of textiles is usually determined by measuring the textiles’ electrical resistance, which is defined as a material’s resistance to the flow of electricity [75]. It is expressed as Ohm per square (Ω/sq), known as a sheet resistance, which is the resistance between two measuring points (electrodes of specific configuration) on a square piece of a fabric surface [70]. In flat textiles, the stability of the electrons makes the flow of electricity difficult, thus electrical resistivity is relatively high (conductivity is relatively low), i.e., the raw cotton fabric shows a resistivity of 10^6^–10^8^ Ω/sq [76]. Instead of sheet resistivity, Ali et al. [77] determined the volume resistivity (in Ω·mm) by applying a voltage potential across opposite sides of the sample and measuring the resultant current flow through the sample.

The electrical conductivity of functionalized flat fabrics (woven or knitted) depends on numerous factors such as the fabric’s structure (density, roughness, volume fraction of pores) [78,79], the uniformity of applied compounds (type and concentration) [41,77], the number of dipping and intermediate drying [77], the measuring method/protocol (measuring line/distance) and the environmental conditions (air humidity and temperature) [70,80]. Tokarska [79] studied the electrical resistivity of circle-shaped woven and square-shaped knitted fabrics in dependence on textiles’ surface roughness. She proved that the smooth surface conducts electrical current better compared with the rough one. Moreover, sample resistance is associated with the density of fabric, i.e., the number of contact points between warp and weft yarns per unit length. Thus, it is important in which testing directions the electrodes are positioned, since the current flows in the path of easiest flow in the fibrous structure.

Ali et al. [77] explained that higher concentration of silver nitrate (AgNO_3_) decreased the conductivity, probably on account of the formation of large Ag particles. Contrarily, lower concentrations of AgNO_3_ (0.10 M) formed the percolated network by creation of continuous connectivity between small silver particles, and thus, developing fabric with high conductivity. At the same time, they found that the greater the number of dips was, the lower was the electrical resistance, due to the denser, greater, and more uniform deposits of conductive particles on the surface. A similar effect was observed by Afroj et al. [41], where the sheet resistance of G-coated poly-cotton fabric was reduced significantly after the second padding pass (from ~32.7 kΩ/sq to ~422 Ω/sq) and continued to decrease steadily up to 5 paddings. Besides an enlarged quantity of G flakes on the fabric surface, the connectivity between the G flakes was improved (the inter-sheet distance between G flakes was reduced) by increasing the number of immersions passes because of the applied compression forces from the padding rollers. Afterwards, the sheet resistance decreased slower as it reached the saturation point. Kazani et al. [80] reported great dependence between water vapor air concentration and electrical resistivity, and, on the other hand, no relation between relative humidity and electrical resistivity, or resistivity and air temperature.

**Figure 5:**
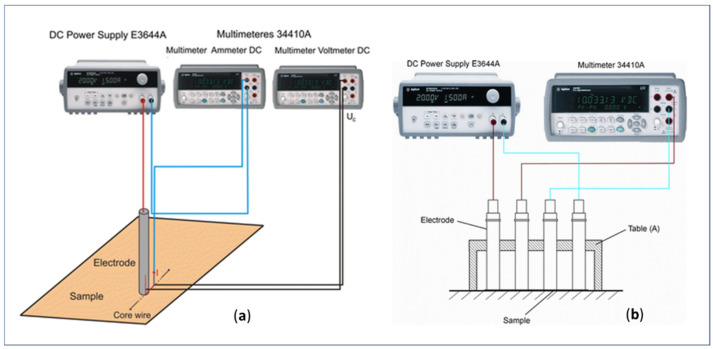
The measuring systems for: (**a**) determination of a contact resistance, and (**b**) multi-variant textile resistance measurements (reprinted under the terms of the CC BY Creative Commons Attribution 4.0 International License from Ref. [78] Copyright 2022 Springer Nature).

Currently, there are no uniform test standards, methods, and/or protocols for measuring the electrical resistance of structurally non-homogeneous textiles (anisotropic electrical resistance). Thus, the comparison between the performance of different electrically conductive flat woven or knitted materials is often difficult and inconsistent [81]. In existing literature, several methods and techniques have been published for measuring the textiles’ surface resistance, such as the two-point probe and the four-point probe (Van Der Pauw method) [75,82,83]. The two-point probe method is the most used because of its simplicity. However, this method can result in measurement error when is utilized on low resistivity materials, due to the influence of additional contact resistance. Compared with the two-point method, the four-point probe measurement gives more accurate results when applied on electrically conductive fabrics. Figure 5 shows a scheme of different measuring systems as described in [78]. Kazani et al. [80] used the so-called “multiple-step method”, which considers the compressional properties of a material, regardless of the mass and shape of samples, providing an indicator inherent for a material’s electrical properties.

However, different textiles-based applications required different levels of electrical conductivity [84,85,86]. Antistatic fabrics, for example, do not need high electrical conductivity to be effective. Typically, a surface resistance of ≤10^5^ Ω/sq is quite sufficient. For EMI shielding applications, resistance should be less than 10^2^ Ω/sq, and applications requiring the transmission of electrical signals suffice a resistance of less than 10^2^ Ω/sq. However, minimal electrical resistance is required for textiles used as wearable electronics, so-called e-textiles or advanced smart textiles, i.e., from 300 Ω/sq to less than 10 Ω/sq.

### 3.2. Morphological and Chemical Properties

With the aim to provide clear and unambiguous information about the fine surface structure of dip-coated electrically conductive textiles, including their chemical composition and interactions, multiple techniques should be employed. 

Scanning electron microscopy (SEM) is the most widely used technique to obtain a detailed surface morphology of textiles and their (un)successful modification at nanoscale level by producing high-resolution surface images [87,88]. Figure 3 shows completely different surface morphology of G nanosheet-coated fabric compared with the morphology of MXene-coated cotton (Figure 4a), influencing diverse textiles´ functionalities. Villanueva et al. [89] used SEM to image cotton thread samples, examine coating uniformity, investigate structural defects during PPy/carbon black composite coating, and determine thread dimensions that have an impact on the properties of wearable conductive textiles. Li et al. [90] found out that lower concentrations of Mxene led to less uniformly coated surface compared with a high concentration (6%). At a higher concentration (8%) the cracks started to show up on the fiber surface, which could affect the electrical conductivity negatively. Mostafalu et al. [14] observed a three-dimensional network of nanofibers on the cotton threads formed by PANI. Importantly, this morphology allows for improved mechanical flexibility along with a more robust coating. In addition, Zheng et al. [67] observed structure variation from the fractioned cross section of the MXene-decorated textile sensor before and after the compression. Xu et al. [43] examined the change of rGO/wool-knitted sensor morphology under the strain. Based on the SEM images, they concluded that the meandering loops formed connections (tentacle-like pathways) to each other in the x direction when being stretched in the y direction. Afroj et al. [41] used the SEM technique to evaluate the washing stability of G flakes on a textile after 5000 washing cycles. 

On the other hand, Ghosh et al. [27] and Berendjchi et al. [91] combined SEM and Atomic Force Microscopy (AFM) in order to evaluate surface topography (i.e., surface roughness) of PEDOT:PSS-coated cotton fabric and RGO/PPy-modified polyester fabric. 

SEM is usually coupled with Energy Dispersive Spectroscopy (EDS) technique (also called Energy-Dispersive X-ray (EDX) spectroscopy), which is used to determine the elemental composition of the inspected samples, and consecutively, to confirm the application of conductive compounds on the surface [92]. 

Other important surface chemistry techniques, which confirmed or disproved SEM results, are X-Ray Powder Diffraction (XRD) for analyzing the crystal structure of modified samples [93], X-Ray Photoelectron Spectroscopy (XPS), which provides information on semi-quantitative elemental analysis and the identification of functional groups [94], and Fourier Transform Infra-Red (FTIR) or Raman spectroscopy, giving insight into the functional groups present on account of dip-coating of conductive compounds through chemical bond vibrations and changes in the local atomic environment [65]. In most research articles dealing with electrically conductive surface modification of textiles, at least one chemical characterization method listed above is applied in combination with SEM, thus, just three examples are presented here. Luo et al. [95] confirmed the successful coating of MXene on the fiber´ surface using XRD. Moreover, the modification steps were followed by XPS and confirmed with specific peaks at binding energies corresponding to MXenes and PDMS. The obtained results were in broad agreement with EDX analysis. Similarly, Li and Du [96] confirmed the presence of PDA on the surface of yarn by means of FTIR spectroscopy. In addition, XRD proved self-reduction of Ag NPs on the MXene surface. In study performed by [65] MXenes-coated cellulose yarns were confirmed by XRD, and XPS analysis was used to track whether the washing process resulted in oxidation or degradation of MXene. 

**Figure 6:**
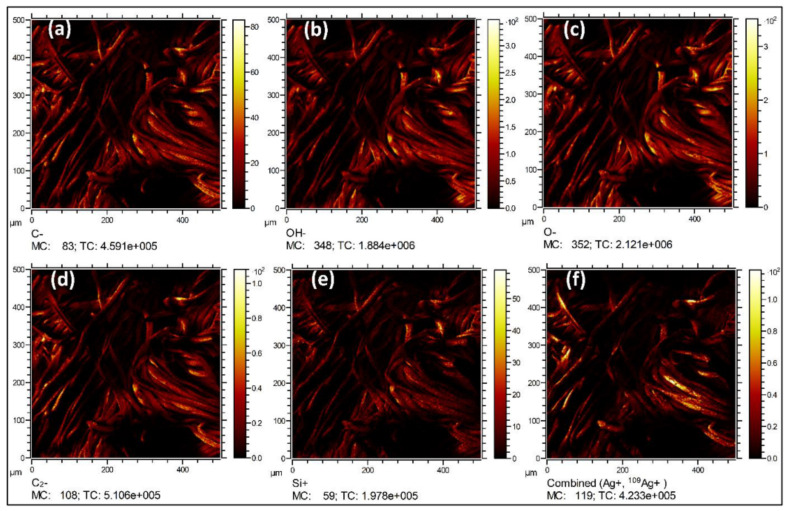
ToF-SIMS mapping of C-CA-RGO-Ag sample: the distribution of (**a**) C^−^, (**b**) OH^−^, (**c**) O^−^, (**d**) C_2_^−^, (**e**) Si^+^, and (**f**) Ag^+^ elements (reprinted with permission from Ref. [33] Copyright 2022 Elsevier).

Besides SEM, FTIR, XRD and XPS, Bhattacharjee et al. [33] reported less frequently employed characterization methods, i.e., Time-of-Flight Secondary Ion Mass Spectrometry (ToF-SIMS) to show the elemental distribution of rGO coated-Ag or rGO/Cu nanoparticle incorporated cotton fabrics (Figure 6), and Optical Emission Spectrometry (OES) to determine the actual amount of the Ag and Cu nanoparticles in samples.

### 3.3. Physical-Mechanical Properties

Since any chemical treatment of textiles changes their physical-mechanical properties to some extent, improving or worsening depending on the type and concentration of compound as well as the process parameters of the dip-coating (number of coating cycles, time and temperature of drying, etc.), it is important that these properties are addressed properly after the application of conductive/functional compounds [97]. It is also expected that the applied compounds adhere tightly to the fiber’s surface, warrantying coating durability [21]. Simultaneously, they should possess flexibility and strength for providing extensibility and protection against external factors. Table 4 includes an overview of recent studies on the testing of mechanical properties of the conductive textiles together with proper standards, if they are stated. The durability against repeated mechanical deformation (bending, compression, and folding) of the conductive fabrics is crucial for their application as strain sensors (strength-strain curves) as emphasized by Sadi et al. [46] in a study dealing with PDA/CNT treated cotton fabric, and Wang et al. [3] fabricating wearable sensing electronics based on MXene-decorated durable cotton fabric (Figure 4b).

Xu et al. [43] reported fabric enhanced tensile stress of rGO-modified knitted wool fabric, which is attributed to the graphene flakes penetrated into the fibers. Goda et al. [99] noticed negligible a reduction of tensile strength and increased elongation of the O-carboxymethyl chitosan-graphene nanosheet/PPy-Ag treated cotton compared with the untreated fabric. The increased elasticity of as-prepared conductive fabric is attributed to the formation of robust hydrogen interaction between the hydroxyl groups of cotton and the functional groups of CMCh on graphene and Ppy-Ag which assist in the effective conduction of stress from the CT chains to the nanocomposite, as well as expediting the polymer crystallization. A similar result was obtained by Ouadil et al. [100] for G/Ag- modified polyester fabric, due to the cross-linking effect between the groups in the fabric, ensuring a more stretchable fabric than those coated with GO and G. As reported by Afroj et al. [41], the hand properties of poly-cotton 2/1 twill fabric (e.g., softness or stiffness and smoothness) determined by the Kawabata evaluation system for fabrics (KES-F) were not deteriorated significantly after applying a fine encapsulation of G flakes. Moreover, G-coated fabric exhibits extremely high bendability, flexibility, and compressibility, with a repeatable response in both forward and backward directions before and after washings.

Berendjchi et al. [91] determined minor differences between the samples´ tenacities, and, on the other hand, significant reduction in the elongation and enlarged bending rigidity of rGO/PPy-treated polyester compared with the pure fabric. The same increase in flexural stiffness was obtained by Lee and Park [101] for PPy-coated cotton fabric. This could be explained by the fact that the PPy deposition increased the fabric’s thickness. With the penetration inside the fabric pores, the PPy formed a rigid conductive layer which restricted the movement of the waft and weft yarns in the fabric structure, making the fabric stiff and inflexible. At the same time, authors reported a decrease in tensile strength of PPy-coated fabric by 16 up to 40% in comparison to the untreated cotton, depending on the oxidant conditions. On the other hand, PPy accompanied with TiO_2_ and TiO_2_/silicon (DTMS) enlarged the tensile strength and elongation at break, as well as decreased the roughness of coated fabrics, as described by Mohamed et al. [102]. 

### 3.4. Comfort Properties (Moisture Absorption, Transport, Thermal Behavior)

The textile material is an interface between the wearer and the environment, and, thus, presents a barrier for heat and vapor transport [103]. The fabric’s structural features, such as weight, thickness, porosity, density, moisture regain, etc., are in correlation with the thermal and water vapor resistance, air permeability, thermal conductivity, and moisture management capacity. The air and moisture permeability of textiles are two of the most important factors affecting the thermo-physiological and sensorial comfort of a wearer (wear comfort of the textile products) [104,105]. Textile air permeability describes the ability of air molecules to pass through the pores of the fabric, while the moisture permeability of textiles describes the ability of water/sweat vapor to pass from the skin to the environment through the garment under transient humidity conditions [45,106,107]. Both parameters are critical in maintaining the thermal balance of the body [108]. Moreover, retained water in the textiles influences their electrical conductivity negatively [43].

Untreated fabrics made from natural fibers (e.g., cotton) are known for their good air permeability, depending on the fabric’s structural parameters. Surface modification and/or the addition of thin layers of nano-compounds usually result in a decrease of air permeability, as also reported by [45,104,109] applying rGO/MXene and carbon nanotube/MXene and PPy, respectively. In a study performed by Zheng et al. [45], the air permeability of cotton fabric was decreased to ae value of 205.5 mm/s after dipping it in a solution of rGO. As explained by the authors, rGO sheets were not only deposited on the surface, but also between the fibers and yarns, blocking the pores of the fabric. Pores’ impermeability for air and moisture is especially pronounced in the case of the employment of conductive polymers, or their combination with other active compounds. In addition, Yu et al. [105] revealed that the moisture content is related negatively to the air permeability of PPy coated cotton fabric, on account of (i) The presence of water, which would make the yarns swell and increase the thickness of the cotton fabric, and (ii) A continuous water film appearance on the surface of the wet fabric, because of surface tension. In their research, the air permeability decreased to almost zero when the water content reached ~80%. 

On the other hand, in a study performed by [107], both air and moisture permeabilities of cotton fabric dip-coated by MXene decreased negligibly, from 1026.6 to 972.2 mm/s, and from 256.54 g/m^2^ to 227.92 g/m^2^, respectively. The authors explained these results by the high affinity between the MXene and cotton fibers, since MXene is coated tightly on the surface of the fibers and does not block the pores between the fibers.

## 4. Wash and Wear Durability

The main obstacle for the mass production of electrically conductive textiles today is the wash and wear durability of conductive compounds; maintaining their electrical performance. During their processing and further over their lifetime, conductive textile-based products are exposed to varying rates of different impacts (environmental, mechanical, and chemical), depending on the final application and intensity of use [110]. The most damaging and destructive forces/conditions act on e-textiles during washing (and drying) cycles (centrifugal forces due to the tumble, squeeze under high temperature, and the addition of detergent/softener), causing oxidation, peeling and/or degradation of the conductive compounds, and, thus, affecting textiles´ conductivity, capacitance, etc. negatively [111,112].

To achieve sufficient durability of the applied coatings, conductive compounds are usually coated or encapsulated with polymer layers [5,43,52], which reduce the electrical conductivity somewhat, as well as form a non-breathable cover that blocks heat and moisture from human skin, as discussed in Section 3.4. Moreover, the protective polymers can impart hydrophobicity, as explained in the last paragraph of Section 5. Mohamed et al. [102] reported excellent electrical conductivity of PPy/TiO_2_/silicon-coated cotton fabric, even after 20 washing cycles. Silicon, in this case, has a role as a cross-linking agent, preventing the peal-off of PPy/TiO_2_ NPs from the fibrous surface, and, thus, maintaining its electro conductive efficacy. Afroj et al. [41] achieved ca. 10-times higher electrical resistance of G-coated and PU encapsulated textiles after 10 washing cycles as compared to G-coated, because PU anchors the G flakes to the textile. Moreover, PU forms a thin and translucent layer around individual G-coated fibers, allowing air to pass through the gap between the fibers, and, thus, enables breathable and comfortable e-textiles. Zheng et al. [67] sandwiched MXene coating between PDMS film and an intermediate electrode, revealing its long-term durability. Zhu et al. [6] reported a slight decrease of electrical conductivity, from 15.1 to 12.5 S/m, after 20 washing cycles (laundry machine, 37 min washing cycle), applying 12 wt% GA/chitosan over SWCNTs-coated textiles.

Instead, employing a cover coating, Sadi et al. [46] increased the adhesion between the fiber’s surface and the loaded CNTs by pre-treatment of the fabric with PDA. The as prepared CNT-PDA-cotton fabric resulted in a conductivity of 41.5 Ω/sq, great durability against repeated mechanical deformation (bending, folding) and multiple washing cycles. Liu et al. [107] dip-coated PEI-pre-treated cotton fabric with Ti_3_C_2_T_x_ in order to deposit a layer of MXene nanosheets uniformly onto the fabric through electrostatic interactions. The developed strain sensor exhibited superior sensitivity (GF = 4.11), a subtle strain detection limit (0.3%), and excellent stability of ~500 washing cycles. 

There are no appropriate standardized methods currently that would prescribe specific conditions and protocols for wash testing of conductive textiles, based on which the comparison of washing performance (electrical resistivity versus the number washing cycles) of diversely coated textiles would be credible [112]. Most researchers who focus on the washability of e-textiles employ existing standards from classical textile manufacturing that do not consider the aspects of integrated electrically conductive components. Moreover, diverse types and quantities of detergents were used, different numbers of cycles, as well as time and temperature [112,113,114]. Additionally, household (machine) washing or simple soaking procedures were described in previous literature. For an example, Lee et al. [114] used an existing AATCC M6 testing Standard to evaluate the washability (four washing cycles) of four types of electrically conductive fabrics made by weaving polyester and nylon yarns with metal coatings (Cu, Ag, Ni/Cu, Ni/Cu/Co) and additionally laminated with thermoplastic polyurethane (TPU). Herein, the AATCC-compliant washing machine and dryer were employed, as well as the controlled variables, i.e., detergent, water temperature, mixing speed, and spin speed, among others. Kisannagar et al. [115], on the other hand, used a simple procedure with 5 g/L of soap in a beaker, stirred for 5 min at 1000 rpm to investigate the adhesion between rGO nanolayers and textiles after one washing cycle.

**Figure 7:**
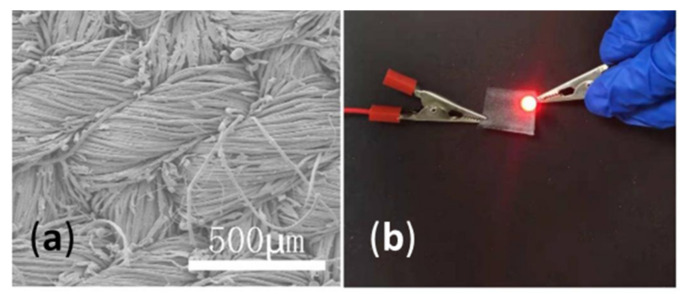
(**a**) SEM micrograph of the SWCNTs/GA-chitosan coated textiles after 1000 cycles of mechanical rubbing using a bristle brush, and (**b**) corresponding electrical conductivity (reprinted with permission from Ref. [6] Copyright 2022 Elsevier).

Another important aspect when using conductive textiles daily is the resistance of the coated compounds against rubbing (fabric to skin, fabric to fabric, fabric to object). Zhu et al. [6] used a multifunction abrasion scrub tester to evaluate the durability of SWCNTs/GA-chitosan coated textiles under wet conditions, employing up to 3,000 rubbing cycles (Figure 7). They concluded that the electrical conductivity of the fabric decreased relatively fast at the beginning (from 7.1 to 4.8 S/m after 100 rubbing cycles), but, after that, slower, (from 4.8 to 4.0 S/m after 3000 rubbing cycles).

In addition, conductive fabrics close to the human body (e.g., wearable sensors) should undergo heat variation, moisture evaporation from metabolic activities, and even immersion with body sweat, i.e., 60 up to 840 mL vapor water/hour, depending on the physical activity [43]. Xu et al. [43] reported quite stable electrical resistance of an rGO/wool-knitted sensor under 20 and 90% of humidity, and a slight decline (11%) of resistance in wet conditions, contracting the fiber laterally upon elongation. Petz et al. [116], on the other hand, exposed the necessity of accelerated functional aging tests to determine the aging behavior of newly developed textile-based sensors. As explained by Biermaier et al. [110] the aging mechanism of e-textiles consists of textile aging (changes in material properties) and the aging of electrical functionalities (losses in conductivity, capacitance, etc.).

## 5. Multifunctionality of Conductive Textiles

With the aim to broaden the application scope of textile-based electronics, and, thus, develop a new generation of safe, all-in-one wearable e-textiles/human-machine interfaces, it is of great importance to combine high electrical conductivity of textiles with their advanced functional features, such as flame retardancy, antibacterial, self-cleaning, UV-shielding ability, thermal stability, hydrophobicity, etc. To attain multi-functionality, numerous compounds can be dip-coated on the fibrous surface, employing: (i) A two- (or more-) steps coating procedure (conductive layer and additional functional layer) or (ii) The combination of conductive and functional compounds simultaneously. Some recent examples of multifunctional conductive textiles utilizing dip-coating are collected in Table 5. 

The UV-shielding ability of knitted and woven fabrics depends on the fabrics´ structure, i.e., the density of threads in both warp and weft directions, the fineness of the yarns, weave type, etc., and finishing treatment, i.e., bleaching process, type of dye and its concentration, etc. [21,124]. It can be enlarged with the addition of special absorbers, such as TiO_2_ nanoparticles, which absorb the damaging UV radiation within a spectral range of 250–400 nm wavelengths and convert it rapidly into harmless thermal energy [125]. Moreover, TiO_2_ NPs show high photocatalytic activity due to their relatively large surface area per unit mass and volume, which enables diffusion of the surface and generates charge-carriers under light irradiation. Thus, Yu et al. [119] (Table 5) reported significantly enhanced Ultraviolet Protective Factor (UPF) of PANI/TiO_2_/cotton, even after 10 washings, depending on the TiO_2_ concentration. The photocatalytic results revealed good absorption and degradation efficiency of PANI/TiO_2_ to rhodamine B (up to 87.67%). Similar UV-protection ability of PPy/TiO_2_/APTEOS/cotton was observed by Mohamed et al. [102]. In addition, they gained good antibacterial efficiency against both gram-positive and gram-negative bacteria. 

The antibacterial activity of conductive textiles is of great importance to eliminate microbial colonization and reduce the possibility of infection, especially when used for sport, military, medical, etc. purposes. Although silver nanoparticles (Ag NPs) themselves own different functional features, such as electrical conductivity, photocatalytic and antibacterial activity, their aggregation tendency and low stability on textiles lead to the reduction of Ag NPs efficiency [100]. Therefore, Ouadil et al. [100] and Makowski et al. [118] combined graphene with Ag NPs for coating of polyester and cotton fabric, respectively, and, thus, increasing the dispersion, stability and activity of Ag NPs. Moreover, graphene particles exhibit antibacterial activity, depending on their dispersibility, adsorption ability, number of corners and sharp edges [118]. Herein, three mechanisms are involved, namely, the action of the sharp edges, oxidative stress, and wrapping or trapping of bacteria due to the flexible thin-film structure of graphene, disrupting the cellular membranes and causing their integrity loss. In addition, Berendjchi et al. [91] studied the antibacterial activity of high molecular weight polymers such as PPy. They can interact with the bacterial cell wall (cytoplasmic membrane) due to the existence of positive charges along the backbone chain, and, thus, eliminate bacteria by leakage of cytoplasm.

**Figure 8:**
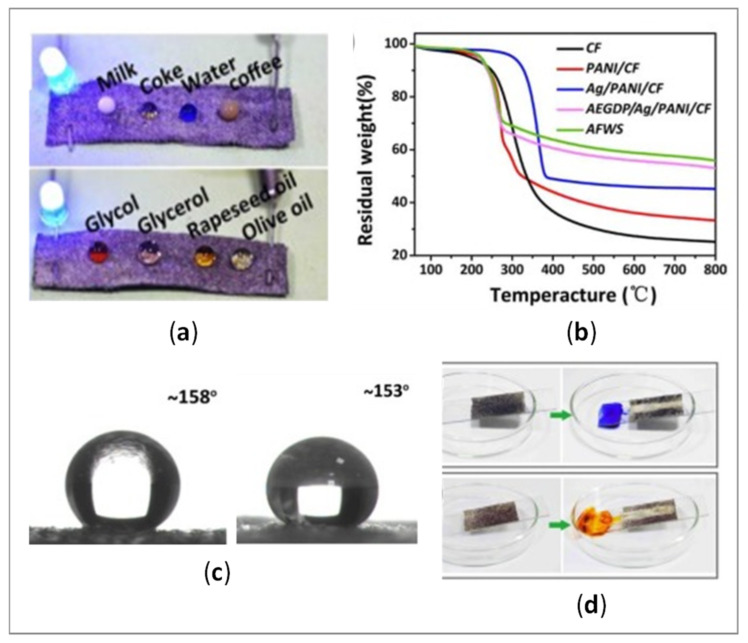
(**a**) Demonstration of Ag/PANI conductive fabric for use in circuits supplying power to LEDs, and corresponding (**b**) TGA results, (**c**) WCA and Oil Contact Angle (OCA) on the surface of samples, and (**d**) The self-cleaning property of the surfaces contaminated with methyl blue and methyl red powders. (Reprinted with permission from Ref. [117] Copyright 2022 Elsevier).

As noticed by Zhao et al. [120] the flame-retardant treatment is almost neglected by researchers dealing with smart textiles, although the high risk of combustion of electrically conductive fabrics exists in the case of unstable resistance, due to the uneven coating. In their study, traditional phosphate flame retardant (PFR) was applied on previously rGO-modified polyester/cotton fabric using the conventional dip-coating procedure. As the result, the Limited Oxygen Index (LOI) value increased from 19 to 32, and the vertical flame test indicated that the damage length reduced to 3.3 cm during the ignition time, while the uncoated fabric burned entirely. Goda et al. [99] observed a more dense, compact, and nonporous char surface when the cotton/polyester blended fabric was coated with O-GRP/PPy-Ag, assisting in the building of a strong insulator layer that prevented the evolution of flammable gases which originated from the textile´s thermal degradation, and, thus, enlarged the flame retardancy. Wang et al. [117] reported a novel, all-weather conductive cellulose fabric with superior electrical stability, even under extreme conditions such as a complex water phase, oil phase, flame and bending, based on Ag/PANI—coating in combination with ammonium salt of ethylene glycol diphosphoric acid (AEGDP) or 1H,1H,2H,2H-perfluorooctyl trichlorosilane (PFOTS)—Figure 8a, as the modified fabric possessed, not only excellent flame retardancy (Figure 8b), but also super amphiphobicity (Figure 8c) and self-cleaning (Figure 8d).

Another highly desired property of conductive textiles is hydrophobicity, which is in close relation with washing repellency, self-cleaning and wear durability [100]. Moreover, a thin layer of hydrophobic compound applied above the conductive elements can protect them from oxidation and degradation due to the action of sweat, water, organic solvents, extreme temperature, etc. [5]. Ouadil et al. [100] reported excellent hydrophobicity (WCA up to of 124°) of a knit polyester fabric coated with graphene/Ag, where graphene nanosheets served as a support to disperse and stabilize the Ag NPs against oxidation. The two main reasons that result in hydrophobic behavior seem to be the removal of hydroxyl groups on the surface of GO nanosheets during drying at 80°, and the removal of oxygen-containing functional groups in the GO during chemical reduction. In research performed by Lee and Park [101], the superhydrophobicity of PPy/DTMS-modified cotton fabric was reached with a contact angle of 150° or higher, and a shedding angle of less than 10° due to the nano-scale roughness caused by the PPy. Similar WCA (151.4°) was attained by Luo et al. [95], depending on the dipping time of PDA/MXene-modified pristine fabric into a PDMS solution. When the dipping time exceeded 40 min, the rough surface became flat due to the thickened PDMS layer, leading to a decrease in WCAs. At the same time, the thick layer of hydrophobic compound applied on the large surface area fills the pores between the fibers, influencing the reduced flexibility and air permeability of textiles, and, consecutively, impairing their comfort properties [5]. Wang et al. [3] noticed that the highly hydrophilic character of MXene can hinder its further application, thus, the fish-scale wrinkles structure 2D d-Ti_3_C_2_T_x_ applied together with Si NPs on the cotton surface enlarged its WCA up to 153° (Figure 8c).

## 6. Sustainability and End-Of-Life of Conductive Textiles

**Figure 9:**
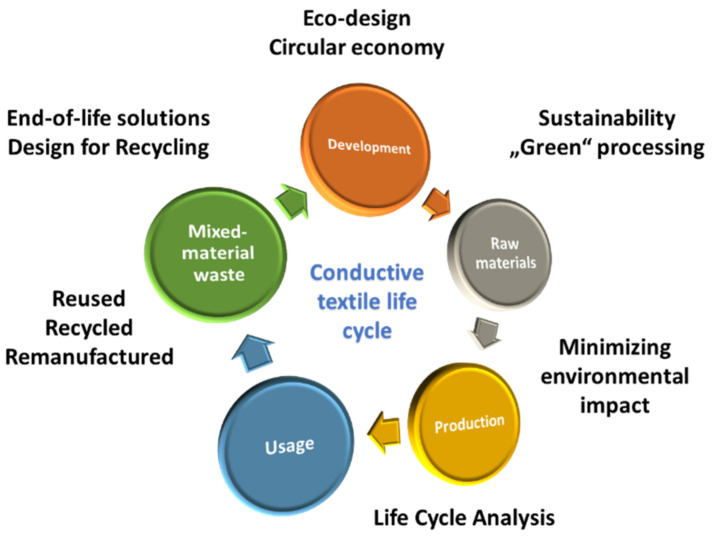
Scheme of life cycle of conductive textiles and their sustainable/recyclable approach.

Another ignored field when dealing with conductive textiles is their sustainable/recyclable aspect (Figure 9), which will become one of the most important parameters for the e-textiles of the future, i.e., usage of natural and/or recycled materials, the “green” processing of the products, and/or the biodegradability or reusability of the components [126,127]. In recent research articles, a maximum of one or two sustainable material/compound and/or process have been used. Ferreira et al. [92] applied Ag NPs on jute fibers via two different green sustainable approaches: Ultraviolet (UV) photoreduction, and by employing polyethylene glycol (PEG) as a reducing agent and stabilizer, attaining relatively low electrical resistivity of 1000 Ω·m. Jagadeshvaran et al. [128] used iron titanate from a sustainable source (ilmenite sand) in combination with MWCNTs for coating of cotton fabric for EMI shielding. Yazıcı et al. [129] developed conductive composites by applying different conductive polymers (PEDOT, PPy, PCz) on reinforced composites of natural fibers from agricultural wastes (artichoke, luffa, banana).

At the end of their rather short lifetime, conductive textiles will unavoidably turn into harder-to-recycle waste, together with built-in contemporary electronic components [130,131]. Currently, there is a lack of standardization of waste streams for e-textiles, and infrastructure does not exist to manage mixed-material waste [131,132]. They may enter the cycling schemes for e-waste (Waste Electrical and Electronic Equipment) or be disposed of together with ordinary old clothes as municipal solid waste (which is more likely). Thus, it is very important to take appropriate measures at the early research stage and find a way in which the environmental impact can be minimized [133].

From a circular economy point of view, the environmental aspects of such products must be considered from the sustainable design (i.e., strategies for disassembly), over sustainability development using bioresources to its End-of-Life (EoL), i.e., e-textiles should be reused, recycled, remanufactured [127,134,135]. Veske and Ilén [136] reviewed current studies and development on the EOL solutions for e-textiles, and Kohler et al. [127] discussed Design for Recycling (DfR) strategies for e-textiles from the background of contemporary innovation trends, focusing on different waste preventative eco-design approaches. Similarly, Schischke et al. [134] provided guidance on how to minimize the environmental footprint of flexible and stretchable smart textiles by analyzing numerous emerging and converging technologies in terms of sustainability implications, depending on material choices and design decisions, although they concluded that more sustainable possibilities are still greatly required, e.g., recycled resources, low-impact processes, and extended products’ lifetimes. Some other studies have focused on Life Cycle Assessment (LCA) of conductive textiles: Radulescu et al. [137] reviewed an LCA analysis of conductive fiber-based flexible electromagnetic shields, and Wójcik-Augustyniak et al. [138] focused on an LCA case study of metallized textiles in the frame of the Maturolife project. Saleem and Zaidi [139] discussed environmental, health, and safety aspects (toxicity) of nano-sized compounds and their sustainable use in different textile applications, due to the wide range of nanomaterials and their properties (shape, size, crystallinity, porosity, agglomeration, and aggregation).

The product eco-design approach follows the principles of the circular economy, considering the same issues and balancing ecological and economic requirements, and, thus, improving textiles’ environmental sustainability [136]. It can be used as a risk mitigation in the design phase. As discussed by Lund et al. [140], the shift towards using non-metallic and bio-based compounds for conductive textiles does fit well with the eco-design approach. Velden et al. [141] reported an LCA case study and eco-design of a wearable smart textile for ambulant medical therapy, focusing on material selection demonstrated through e-textile product redesign, as one of the most relevant choices at the prototyping stage. Lacruz et al. [142] synthesized a series of waterborne polyurethane-urea dispersions under strict sustainability and eco-design criteria, which were further doped with SWCNTs for electrically conductive and hydrophobic textile coatings.

## 7. Conclusions

The inclusion of electrical conductivity into flexible fiber-forming materials is a promising research topic, leading to the fabrication of wearable textile-based electronics with applications such as electronic sensors, energy harvesting, data storage, EMI shielding, wireless communication, personal thermal management, light emitting, tracking, etc. Among the diverse methods used to obtain electro conductive textiles, the dip-coating technique has received enormous attention due to its relative simplicity, low-cost, and ease of integration into existing textile finishing plants. Due to the numerous conductive compounds and auxiliaries, diverse raw materials, pre-treatment processes, different parameters of dip-coating procedures and different measuring devices and protocols, it is hard to compare as-prepared conductive textiles with each other in terms of coating efficiency. Moreover, different types of conductive compounds are frequently combined in one coating formulation (or a conductive compound with other functional nanoparticles to attain textiles’ multifunctionality), which can, contradictorily, influence the physical-mechanical, optical, and comfort properties of textiles as well as on compounds’ durability.

Conductive textiles are usually in close contact with skin to collect data, and, thus, subjected to different repeatable extreme conditions during wear (sweat, temperature, rubbing, etc.) and further care (water, chemicals, mechanical forces, etc.), which can cause oxidation and/or peel-off the compounds. Consequently, their conductivity decreases with time, which is the main reason for the lack of commercially available e-textiles. The adhesion and bonding strength of conductive compounds can be enhanced by the proper pre-treatment and/or application of additional protective layers, usually polymers. On the other hand, particularly conductive polymers can block the pores between the fibers in textiles when applied in the form of a thick layer (or several thin layers), influencing the textiles’ permeability for air and moisture. This can affect the thermo-physiological and sensorial comfort of the wearer, especially when a larger surface area is coated. 

Based on the above, an interdisciplinary approach is essential for studies on conductive textiles, where strong collaboration between different discipline teams is required, i.e., textile and polymer, materials science, electrochemistry, nanotechnology, and characterization, with a special focus on sustainability over the whole product cycle, from the design and development, through processing, energy and water supply chain, and recyclability at the end of the product’s lifetime. Moreover, the existing dip-coating procedure should be improved in terms of the adhesion and durability of compounds, and proper protocols and standards should be introduced for the washability and aging testing of conductive textiles.

## Figures and Tables

**Table 1:** Review of completed research projects based on selected key words and their combinations.

Key Words	Projects		
	FP6	FP7	H2020
“electronic textile”	0	0	4
“e-textile”	2680	6214	9702
“conductive textile”	0	0	2
“smart textile”	3	14	21
“electronic” AND “textile”	7	24	98
“conductive” AND “textile”	8	16	56

**Table 2:** Dip-coating of electro-conductive polymers on textiles for diverse applications.

Textile	Conductive Layer	Electrical Resistance	Electrical Conductivity	Application	Reference
aramid fabric	PEDOT:PSS PANI:CSA ^1^		0.61 mS/cm 1.8 · 10^−4^ mS/cm	radar absorbing structure	[26]
cotton fabric	PEDOT:PSS		51–82.7 S/cm	EMI shielding	[27]
cotton, rayon and polyester	PANI	14.7 ± 4.8 kΩ/cm		gas sensors for real-time monitoring of ammonia gas	[28]
polyamide/lycra knitted fabric	PEDOT:PSS	1.7 Ω/sq	14 S/cm	wearable e-textiles	[24]
silk yarn	PEDOT:PSS		70 S/cm	touch sensor device	[29]
cotton, wool, silk and polyester	PPy	˂10 Ω/sq		wearable heater	[30]
polyester fabric	PEDOT:PSS		1.5 S/cm	energy harvesting	[31]

^1^ camphorsulfonate (CSA).

**Table 3:** Dip-coating of carbon-based materials on textiles for diverse applications.

Textile	Conductive Layer	Electrical Resistance	Electrical Conductivity	Application	Reference
cotton fabric	rGO	13.8 ± 2.7 Ω/sq	580.1 ± 4.3 S/m	electrochemical energy storage, EMI shielding, electrothermal and human motion detection	[45]
nylon and cotton	GO	350 Ω/sq 1 kΩ/sq		temperature, humidity, and heart rate sensors	[38]
polyurethane yarn	rGO			strain sensing	[44]
nylon 6 filaments	G		6.43 S/m	strain sensing	[39]
knitted para-aramid fabric	G	7.5 × 10^4^ Ω/sq		wearable heater	[22]
knitted wool fabric	rGO			strain sensing	[43]
spandex yarn	G		up to 100 S/m	health monitor sensing	[40]
cotton fabric	SWCNT	555 Ω/sq		wearable electronics and heater	[46]
nylon spandex fabric	MWCNT and rGO			strain sensing	[36]
cotton fabric	MWCNT G	33.2 Ω/sq 29.8 Ω/sq		strain sensing and wearable heater	[42]
polyamide fabric	SWCNT		up to 7.4 × 10^2^ S/m	EMI shielding and wearable heater	[6]

**Table 4:** Overview of mechanical properties’ testing.

Textile	Conductive Layer	Mechanical Property	Standard	Reference
Cotton fabric	Co, Cu, Mn or PANI	Tensile strength	ASTM D 5034	[98]
		Elongation at break		
Knitted wool fabric	rGO	Tensile strength	-	[43]
Cotton/polyester fabric	O-carboxymethyl chitosan-graphene nanosheet/PPy-Ag	Tensile stress	-	[99]
		Elongation at break		
Polyester fabric	rGO/PPy	Tensile properties	ASTM D 5035	[91]
		Bending rigidity	ASTM D 1388-96	
Polyester fabric	G, GO, G/Ag	Tensile strength	-	[100]
		Elongation at break		
Cotton fabric	PPy	Tensile strength	ASTM D 5035	[101]
		Stiffness	ISO 4606: 2013	
Cotton fabric	PPy	Tensile strength	ASTM D 1682-2004	[102]
		Elongation at break		

**Table 5:** Overview of multifunctional electro-conductive textiles utilizing the dip-coating technique.

Textile	Coating Layer	Functional Features	Reference
Cellulose fabric	Ag/PANI/AEGDP ^1^ Ag/PANI/PFOTS ^2^	flame retardancy, super amphiphobicity and self-cleaning	[117]
Cotton yarn	CNT/PPy	antibacterial	[73]
Polyester fabric	rGO/PPy	antibacterial, UV-protective and thermal stability	[91]
Cotton fabric	rGO/Ag	antibacterial	[118]
Cotton fabric	PANI/TiO_2_	UV-protective, photocatalytic activity and thermal stability	[119]
Cotton fabric	Ppy/TiO_2_/APTEOS ^3^	UV-protective and antibacterial	[102]
Cotton/polyester fabric	O-GRP ^4^/PPy-Ag	antibacterial and flame retardancy	[99]
Cotton/polyester fabric	rGO/PFR ^5^	flame retardancy and thermal stability	[120]
Polyester fabric	G, GO, G/Ag	antibacterial, hydrophobic and thermal stability	[100]
Cotton and polyester fabrics	PANI	antibacterial	[121]
Cotton fabric	PPy/DTMS ^6^	superhydrophobic and self-cleaning	[101]
Cotton fabric	MWCNTs–GPTMS ^7^/tannic acid	antibacterial and UV-protective	[122]
Pristine fabric	PDA ^8^/MXene/PDMS	superhydrophobic	[95]
Cotton fabric	MWCNTs/stearoyl chloride	superhydrophobic and thermal stability	[123]

^1^ ammonium salt of ethylene glycol diphosphoric acid (AEGDP); ^2^ 1H,1H,2H,2H-perfluorooctyl trichlorosilane (PFOTS); ^3^ 3-Aminopropyltriethoxysilane (APTEOS); ^4^ O-carboxymethyl chitosan—graphene nanosheet; ^5^ phosphate flame retardant (PFR); ^6^ dodecyltrimethoxysilane (DTMS); ^7^ 3-glycidoxypropyltrimethoxy silane (GPTMS); ^8^ polydopamine (PDA).

## Data Availability

Not applicable.
